# VTE Registry: What Can Be Learned from RIETE?

**DOI:** 10.5041/RMMJ.10171

**Published:** 2014-10-29

**Authors:** Inna Tzoran, Benjamin Brenner, Manolis Papadakis, Pierpaolo Di Micco, Manuel Monreal

**Affiliations:** 1 Department of Hematology and Bone Marrow Transplantation, Rambam Health Care Campus, Haifa, Israel;; 2 Bruce Rappaport Faculty of Medicine, Technion, Israel Institute of Technology, Haifa, Israel;; 3 Department of Hematology, Hospital Papageorgion, Saloniki, Greece;; 4 Department of Internal Medicine, Ospedale Buon Consiglio Fatebenefratelli, Naples, Italy; 5 Department of Internal Medicine, Hospital Universitari Germans Trias i Pujol, Badalona, Spain

**Keywords:** Anticoagulant therapy, outcome, registry, venous thromboembolism

## Abstract

The Registro Informatizado de Enfermedad TromboEmbólica (RIETE Registry) is an ongoing, international, prospective registry of consecutive patients with acute venous thromboembolism (VTE) designed to gather and analyze data on treatment patterns and outcomes in patients with acute VTE. It started in Spain in 2001, and 6 years later the database was translated into English with the aim to expand the Registry to other countries. In contrast to randomized controlled trials, there is no imposed experimental intervention: the management is determined solely by physicians. Thus, it provides data on patients with VTE in a real-world situation with an unselected patient population. Data from RIETE are hypothesis-generating and provide feedback from real-world clinical situations. So far, we learned about the natural history of VTE in patients with relative or absolute contraindications to anticoagulant therapy. We also learned interesting aspects on the natural history of VTE, and we built a number of prognostic scores to identify VTE patients at low, moderate, or high risk for adverse outcome.

## INTRODUCTION

Current guidelines on antithrombotic therapy provide a critical review of the literature related to the management of patients with venous thromboembolism (VTE) and lay the scientific groundwork for the standard of care, based largely on data from randomized controlled clinical trials.^1^ However, a number of VTE patients are often excluded from randomized clinical trials (for example due to pregnancy, renal insufficiency, or high risk of bleeding). Thus, there is little evidence on what would be the best therapeutic approach for these patients.

The Registro Informatizado de Enfermedad TromboEmbólica (RIETE Registry) is an ongoing, international (Spain, Italy, France, Israel, Portugal, Germany, Switzerland, Belgium, Czech Republic, Republic of Macedonia, Greece, Canada, Brazil, United States, Argentina, and Ecuador), prospective registry of consecutive patients with acute VTE designed to gather and analyze data on treatment patterns and outcomes in patients with acute VTE. It started in Spain in 2001, and 6 years later the database was translated into English with the aim to expand the Registry to other countries, ultimately allowing physicians worldwide to use the database. Our aim was to improve the treatment of VTE through a better understanding of patient demographics, management, and in-hospital and post-discharge outcomes.

In contrast to a randomized controlled trial, there is no imposed experimental intervention: the management is determined solely by physicians. Thus, it provides data on patients with VTE in a real-world situation with an unselected patient population. It can, therefore, help to identify practices for providing treatment to patients, and factors associated with better or worse patient outcomes. Data from RIETE are hypothesis-generating and provide feedback from real-world clinical situations which may be of help when designing new randomized clinical studies.

### Data Collection and Monitoring

Participating physicians ensure that eligible patients are consecutively enrolled. Data are recorded onto a computer-based case report form at each participating hospital and submitted to a centralized coordinating center through a secure website. The study coordinating center assigns patients with a unique identification number to maintain patient confidentiality and is responsible for all data management. Data quality is regularly monitored electronically, including checks to detect inconsistencies or errors, which are resolved by contacting the local coordinators. Data quality is also monitored by periodic visits to participating hospitals by contract research organizations that compare the information in medical records with the submitted data in the website.

### Inclusion Criteria

Consecutive patients with acute deep vein thrombosis (DVT) or pulmonary embolism (PE), confirmed by objective tests, are enrolled. Patients are excluded only if they are currently participating in a therapeutic clinical trial with a blind medication. All patients provided informed consent to their participation in the Registry, according to the requirements of the ethics committee within each hospital.

### Treatment and Follow-up

Patients are managed according to the clinical practice of each participating hospital (i.e. there is no standardization of treatment). The drug, dose, and duration of anticoagulant therapy are recorded. During each visit, any signs or symptoms suggesting VTE recurrences, bleeding complications, and any other adverse events are noted. After initial diagnosis, patients are followed up for at least 3 months, but there is no limit for the duration of follow-up. In 2010 we requested all the members to prolong the follow-up in all their patients for at least 12 months. In December 2013, there are over 10,000 VTE patients followed up for 1 year, and over 5,000 for 2 years.

## STUDY SUPPORT

The RIETE Registry is an independent registry, supported by Sanofi-Aventis in Spain and by Bayer Pharma AG outside Spain. During the first 5 years, RIETE was also supported by Red Respira from the Instituto Carlos III, Spain (Red Respira-ISCiii-RTIC-03/11). Neither Sanofi-Aventis nor Bayer Pharma AG have any right of access to the database, and there is no payment per recruited patient.

### Management of VTE in Patients with Special Conditions

In a first article using the RIETE database, we found that 26% of patients with VTE had at least one exclusion criterion to be recruited in randomized clinical trials of antithrombotic therapy. Interestingly, these patients had a 4-fold higher rate of fatal PE and a 4-fold higher rate of fatal bleeding than VTE patients with no exclusion criteria.^1^

We assessed the outcome of VTE patients with recent (<30 days earlier) major bleeding. Of 6,361 patients enrolled by 2004, 170 had experienced recent major bleeding: 41% in the gastrointestinal tract (GI), 35% intracranial, 24% other. On multivariate analysis, the odds ratios (OR) for fatal bleeding (6.4; 95% CI 2.6–15) and fatal recurrent PE (4.5; 95% CI 1.3–14) were significantly higher in patients with recent major than in those without recent bleeding.^2^ In a further study with 306 patients, we found that patients with recent GI bleeding had an increased risk for both major re-bleeding (hazard ratio [HR] 2.8; 95% CI 1.4–5.3) and death (HR 1.9; 95% CI 1.2–3.1) compared to those with no recent bleeding. Those who bled in other sites had an increased risk only for death (HR 2.0; 95% CI 1.2–3.3). Moreover, an elapsed time of <2 weeks from bleeding to the index VTE event was also associated with an increased risk for major re-bleeding (HR 2.4; 95% CI 1.2–5.0) and death (HR 2.8; 95% CI 1.8–4.5).^3^

Of 13,011 patients enrolled by 2005, 2,890 (22%) were aged >80 years. During the first 3 months of therapy, 99 patients (3.4%) aged >80 years and 212 aged <80 years (2.1%) had major bleeding (OR 1.7; 95% CI 1.3–2.1).^4^ Fatal bleeding occurred in 0.8% and 0.4%, respectively (OR 2.0; 95% CI 1.2–3.4). The rate of recurrent VTE was 2.1% and 2.8%, but 3.7% of patients >80 years and 1.1% <80 years died of PE (OR 3.6; 95% CI 2.7–4.7). In another study, of 21,873 patients enrolled by 2008, 610 (2.8%) were aged ≥90 years.^5^ During the 3-month study period, 140 patients (23%) died: 45 patients died of PE, 18 died of bleeding. On multivariate analysis, fatal PE was significantly more common in patients with recent immobility. Finally, we assessed the risk of fatal PE and fatal bleeding in 16,199 patients with lower limb DVT (without symptomatic PE at the time of inclusion), with patients categorized according to age.^6^ During the first 3 months of treatment, there were 31 fatal PE (0.19%) and 83 fatal bleeds (0.51%). During the first 7 days of therapy, the frequency of fatal PE was similar to that of fatal bleeding (12 versus 14 deaths, respectively; OR 0.86; 95% CI 0.39–1.87). However, from Day 8 to Day 90 the frequency of fatal bleeding was greater than that of fatal PE (69 versus 19 deaths; OR 3.64; 95% CI 2.22–6.20). The higher frequency of fatal bleeding compared to fatal PE from Day 8 to Day 90 appeared to be confined to patients aged ≥60 years. On multivariate analysis, the patient’s age was independently associated with an increased risk for death from bleeding during the first 3 months: every 10 years the odds ratio increased by 1.37 (95% CI 1.12–1.67).

A total of 14,391 patients were enrolled by 2006. Of these, 2,945 (20%) had active cancer.^7^ During the first 3 months, the rate of fatal PE in cancer patients was 2.6%, and that of fatal bleeding 1.0%. These rates were significantly higher than in patients without cancer (1.4% and 0.3%, respectively). Renal insufficiency, metastatic disease, recent major bleeding, and recent immobility (42% of the 108 patients who died from PE or bleeding had recent immobility) were independently associated, in patients with cancer, with an increased risk for both fatal PE and fatal bleeding. In 2007, we built a clinical prediction rule to identify which cancer patients were at a higher risk for recurrent PE or major bleeding during the first 3 months of anticoagulation.^8^ On multivariate analysis, patients aged <65 years, with clinically overt PE at baseline, or with <3 months from cancer diagnosis to VTE had an increased incidence of recurrent PE. A risk score was calculated: the incidences of recurrent PE in patients with low, intermediate, or high risk were 0.6%, 2.5%, and 7.4%, respectively. Patients with immobility ≥4 days, severe renal insufficiency, metastases, or recent bleeding had an increased incidence of major bleeding. The incidences of major bleeding in patients with low, intermediate, or high risk were 1.8%, 5.0%, and 15%, respectively, and their positive likelihood ratios were 0.44, 1.22, and 4.02.

In another study, we compared the outcome of cancer patients with VTE according to their white blood cell (WBC) count at baseline.^9^ Of 3,805 patients enrolled, 5.7% had low, 63% normal, and 31% elevated WBC count. During the first 3 months, patients with elevated WBC count had an increased incidence of recurrent VTE (OR 1.6; 95% CI 1.2–2.2), major bleeding (OR 1.5; 95% CI 1.1–2.1), or death (OR 2.7; 95% CI 2.3–3.2). On multivariate analysis, patients with elevated WBC count were at increased risk for all three complications.

In patients with extreme body weight, we found no differences in outcome between those weighing 50–100 kg or over 100 kg, but patients weighing <50 kg had a significantly higher rate of bleeding complications.^10^ Then, we examined the association between body mass index (BMI) and mortality during the first 3 months of therapy.^11^ Of the 10,114 patients, 1.5% were underweight (BMI <18.5), 28% normal weight (BMI 18.5–24.9), 43% overweight (BMI 25.0–30), and 27% were obese (BMI >30). Obese patients had less than half the mortality than normal BMI patients. This reduction in mortality rates was consistent among all subgroups and persisted after multivariate adjustment.

Of 10,526 patients enrolled in 2005, 88% had creatinine clearance (CrCl) levels >60 mL/min, 6.7% had 30–60 mL/min, and 5.6% had CrCl <30 mL/min.^12^ The rate of fatal PE during the first 15 days was 1.0%, 2.6%, and 6.6%, respectively. Fatal bleeding occurred in 0.2%, 0.3%, and 1.2%, respectively. Thus, patients with renal insufficiency had an increased rate of both fatal PE and fatal bleeding, but the risk of fatal PE far exceeded that of fatal bleeding. Our data supported the use of full-dose anticoagulant therapy, even in patients with severe renal insufficiency. In another study, we evaluated the 15-day outcome in 38,531 patients and used propensity score matching to compare patients treated with unfractionated heparin (UFH) with those treated with low-molecular-weight heparin (LMWH) in three groups stratified by CrCl levels at baseline: >60 mL/min, 30–60 mL/min, or <30 mL/min.^13^ Propensity score-matched groups showed an increased 15-day mortality for UFH compared with LMWH (4.5% versus 2.4% [*P* < 0.001], 5.4% versus 5.8% [*P* = NS], and 15% versus 8.1% [*P* = 0.02]), an increased rate of fatal PE (2.8% versus 1.2% [*P* < 0.001], 3.2% versus 2.5% [*P* = NS], and 5.7% versus 2.4% [*P* < 0.02]), and a similar rate of fatal bleeding (0.3% versus 0.3%, 0.7% versus 0.7%, and 0.5% versus 0.0%). Multivariate analysis confirmed that patients treated with UFH were at increased risk for all-cause death (OR 1.8; 95% CI 1.3–2.4) and fatal PE (OR 2.3; 95% CI 1.5–3.6).

In pregnant patients, we found that VTE developed during the first trimester in 40%, thus suggesting that VTE prophylaxis, when indicated during pregnancy, should start in the first trimester.^14^ Interestingly, no patient recurred or bled before delivery, but after delivery the risk of major bleeding (5.6%) exceeded the risk of recurrences (1.4%).

### Natural History of VTE in Non-selected Patients

Since we gathered data on consecutive patients with VTE, we assessed the natural history of VTE in unselected patients, and compared their clinical characteristics and outcome. We found that acutely ill medical patients with immobility ≥4 days had a more severe VTE presentation than postoperative patients, and that the incidence of fatal PE and fatal bleeding were much higher in immobilized patients than in the surgical.^15^ Then, we analyzed the clinical characteristics, time-course, and 3-month outcome of all patients with postoperative VTE. Of these, 25% had major orthopedic surgery, 13% cancer surgery, and 63% had other procedures. Their clinical presentation, time-course, and 3-month outcome was similar, but the use of prophylaxis in patients undergoing cancer surgery or other procedures was suboptimal. We also studied the natural history of patients with upper-extremity DVT.^16^ Of the 11,564 DVT patients enrolled in 2007, 512 patients (4.4%) had arm DVT. They presented less often with clinically overt PE (9.0% versus 29%) than those with lower-limb DVT, but their 3-month outcome was similar.

In two studies, we compared the clinical characteristics and outcome of patients with a first episode of VTE and factor V Leiden, prothrombin G20210A, or no thrombophilia.^17^,^18^ In 2009, 345 patients had factor V Leiden, 261 had prothrombin G20210A, and 2,399 tested negative. Sixty-two percent of VTE episodes in women (40% in men) were associated with an acquired risk factor. Among women with factor V Leiden or prothrombin G20210A, contraceptive use and pregnancy accounted for 63% and 67% of such risk factors. Patients with factor V Leiden presented with PE less often (31% versus 51% or 45%), and less likely had hypoxemia (4.5% versus 17% and 20%). There were no differences between subgroups in the incidence of recurrent VTE, either during or after discontinuation of anticoagulant therapy. We also studied the natural history of VTE in patients with chronic lung disease,^19^–^21^ in women using hormonal therapy,^22^,^23^ and in patients with distal DVT, among others.^24^

### Prognostic Scores

We built a score to predict the risk for major bleeding within the first 3 months of anticoagulant therapy (Table 1).^25^ Of 19,274 patients enrolled, 13,057 were randomly assigned to the derivation sample, 6,572 to the validation sample. On multivariate analysis, age >75 years, recent bleeding, cancer, creatinine levels >1.2 mg/dL, anemia, or PE at baseline were independently associated with an increased risk for major bleeding. A score was composed assigning 2 points to recent bleeding, 1.5 to abnormal creatinine levels or anemia, 1 point to the remaining variables. In the derivation sample 20% of patients scored 0 points; 74% scored 1–4 points; and 5.8% scored >4 points. The rates of major bleeding were: 0.3% (95% CI 0.1–0.6), 2.6% (95% CI 2.3–2.9), and 7.3% (95% CI 5.6–9.3), respectively. The likelihood ratio tests were 0.14 (95% CI 0.07–0.27) for patients at low risk and 2.96 (95% CI 2.18–4.02) for those at high risk. In the validation sample the incidences of major bleeding were 0.1%, 2.8%, and 6.2%, respectively.

**Table 1.** Prognostic Score for Major Bleeding.

	Odds Ratio (95% CI)	*P* Value	Points
Recent major bleeding	2.7 (1.6–4.6)	<0.001	2
Creatinine levels >1.2 mg/dL	2.1 (1.7–2.8)	<0.001	1.5
Anemia	2.1 (1.7–2.7)	<0.001	1.5
Cancer	1.7 (1.4–2.2)	<0.001	1
Clinically overt PE	1.7 (1.4–2.2)	<0.001	1
Age >75 years	1.7 (1.3–2.1)	<0.001	1

We also assessed risk factors for fatal bleeding (Table 2).^26^ Of 24,395 patients, 2.24% had a major bleed and 0.55% had a fatal bleed during the first 3 months of anticoagulation. Fatal bleeding was independently associated with the following factors at the time of VTE diagnosis: age >75 years, metastatic cancer, immobility, a major bleed within the past 30 days, an abnormal prothrombin time, a platelet count <10^9^/L, CrCl levels <30 mL/min, anemia, and distal DVT. A clinical prediction rule for risk of fatal bleeding that included these nine baseline factors was derived. Fatal bleeding occurred in 0.16% (95% CI 0.11–0.23) of the low-risk, 1.06% (95% CI 0.85–1.30) of the moderate-risk, and 4.24% (95% CI 2.76–6.27) of the high-risk category. Some years later we validated this score in a further sample of patients.^27^

**Table 2.** Prognostic Score for Fatal Bleeding.

	**Odds Ratio**	**95% CI**	**Points**
Age >75 years	2.16	1.49–3.16	1
Recent major bleeding	2.64	1.44–4.83	1.5
Immobility ≥4 days	1.99	1.40–2.83	1
Metastatic cancer	3.80	2.56–5.64	2
Anemia	1.54	1.07–2.22	1
Platelet count <10^9^/L	2.23	1.16–4.29	1
Abnormal prothrombin time	2.09	1.34–3.26	1
CrCl levels <30 mL/min	2.27	1.49–3.44	1
Distal DVT	0.39	0.16–0.95	−1

In another study, we compared the pulmonary embolism severity index (PESI) clinical prediction rule for estimating the risk of 30-day mortality in patients with PE with a simplified PESI (sPESI) in a derivation cohort of Spanish patients (Table 3).^28^ Then, we used the RIETE database to validate externally the sPESI score. In the derivation data set, univariate logistic regression of the original 11 PESI variables led to the removal of variables that did not reach statistical significance and subsequently produced the sPESI that contained the variables of age, cancer, chronic cardiopulmonary disease, heart rate, systolic blood pressure, and oxyhemoglobin saturation levels. The prognostic accuracy of the original and simplified PESI scores did not differ (area under the curve, 0.75, 95% CI 0.69–0.80). The 305 of 995 patients (30.7%) who were classified as low risk by the simplified PESI had a 30-day mortality of 1.0% (95% CI 0.0%–2.1%) compared with 10.9% (95% CI 8.5%–13.2%) in the high-risk group. In the RIETE validation cohort, 2,569 of 7,106 patients (36.2%) who were classified as low risk by the simplified PESI had a 30-day mortality of 1.1% (95% CI 0.7%–1.5%) compared with 8.9% (95% CI 8.1%–9.8%) in the high-risk group.

**Table 3.** Original Pulmonary Embolism Severity Index (PESI) and Simplified PESI Scores.

	Original PESI^*^	**Simplified PESI^†^**
Age >80 years	Age in years	1
Male sex	+10	
History of cancer	+30	1
History of heart failure	+10	1 or ^‡^
History of chronic lung disease	+10	or 1
Pulse ≥110 beats per minute	+20	1
Systolic blood pressure <100 mmHg	+30	1
Respiratory rate ≥30 breaths per minute	+20	
Temperature <30ºC	+20	
Altered mental status	+60	
Arterial oxyhemoglobin saturation <90%	+20	1

* A total point score for a given patient is obtained by summing the patient’s age in years and the points for each predictor when present. The score corresponds with the following risk classes: 65 or less, class I; 66–85, class II; 86–105, class III; 106–125, class IV; and more than 125, class V. Patients in risk classes I and II are defined as being at low risk.

† A total point score for a given patient is obtained by summing the points. The score corresponds with the following risk classes: 0, low risk; 1 or more, high risk. Empty cells indicate that the variable was not included.

‡ The variables were combined into a single category of chronic cardiopulmonary disease.

We also produced prognostic scores to identify outpatients with DVT at low risk for complications,^29^ cancer patients at increased risk of dying,^30^ predictors for fatal PE,^31^ or to identify cancer patients with PE at low risk for complications.^32^

### Limitations and Strengths

The RIETE Registry has several limitations. First, patients are not treated with a standardized anticoagulant regimen; treatment varies with local practice and is likely to be influenced by a physician’s assessment of a patient’s risk of bleeding. Second, to fulfil the definition of fatal PE in RIETE, patients must first experience an objectively confirmed PE event, followed by death within 10 days. Thus, all sudden unexplained deaths which are usually considered as “likely” fatal recurrent PE are not considered, and the rate of fatal PE may have been underestimated, especially after hospital discharge. Third, RIETE is an ongoing observational registry (and not a randomized controlled trial), and the data are hypothesis-generating. Thus, we should be extremely cautious before suggesting changes in treatment strategies based on uncontrolled registry data. Finally, there is no monitoring and no external control of the data entered in RIETE, and there is also no external adjudication of the events, which are merely reported by the authors. Strengths of the Registry include that a large number of consecutive unselected patients are enrolled, and a large number of variables are considered.

## CONCLUSIONS AND FUTURE PERSPECTIVES

Our data suggest that the information gathered in RIETE may be a useful basis for future controlled clinical trials investigating modified anticoagulant regimens versus standard therapy in special VTE populations. Moreover, our data provide information on the epidemiology of VTE in different countries, and might be useful to monitor the efficacy and safety of the new anticoagulants in the near future.

## Abbreviations:

BMI: body mass index;
CrCl: creatinine clearance;
DVT: deep vein thrombosis;
GI: gastrointestinal tract;
HR: hazard ratio;
LMWH: low-molecular-weight heparin;
OR: odds ratio;
PE: pulmonary embolism;
PESI: pulmonary embolism severity index;
RIETE: Registro Informatizado de Enfermedad TromboEmbólica;
sPESI: simplified pulmonary embolism severity index;
UFH: unfractionated heparin;
VTE: venous thromboembolism;
WBC: white blood cell.
